# The effect of the Alive & Thrive initiative on exclusive breastfeeding in rural Burkina Faso: a repeated cross-sectional cluster randomised controlled trial

**DOI:** 10.1016/S2214-109X(18)30494-7

**Published:** 2019-02-14

**Authors:** Jenny A Cresswell, Rasmané Ganaba, Sophie Sarrassat, Henri Somé, Abdoulaye Hama Diallo, Simon Cousens, Veronique Filippi

**Affiliations:** a MARCH Centre for Maternal, Adolescent, Reproductive & Child Health, London School of Hygiene & Tropical Medicine, London, UK; b AFRICSanté, Bobo-Dioulasso, Burkina Faso; c Centre MURAZ, Bobo-Dioulasso, Burkina Faso; d Université Ouaga I Pr Joseph Ki-Zerbo, Ouagadougou, Burkina Faso

## Abstract

**Background:**

The benefits of exclusive breastfeeding on mortality, health, and development of children have been well documented. In Burkina Faso, the Alive & Thrive initiative combined interpersonal communication and community mobilisation activities with the aim of improving knowledge, beliefs, skills, and, ultimately, breastfeeding outcomes. The objective of this study was to determine the effect of the Alive & Thrive initiative on exclusive breastfeeding in Boucle du Mouhoun, Burkina Faso.

**Methods:**

We did a cluster-randomised trial with data collected with two independent, population-representative, cross-sectional surveys: a baseline survey done before the start of the initiative implementation and an endline survey done 2 years later. Rural villages in Boucle du Mouhoun, Burkina Faso, were randomly allocated by use of computer generated pseudo-random numbers, and women were eligible for participation if they had a livebirth in the 12 months preceding the survey and resided in a village selected for the study. The primary outcome was exclusive breastfeeding among infants younger than 6 months. Masking was not possible for the intervention implementation. All women who participated in the trial were included in the analysis population. The trial is registered with ClinicalTrials.gov, number NCT02435524.

**Findings:**

Between June 2 and July 28, 2015, 2288 mothers participated in the baseline survey and between June 12 and July 25, 2017, 2253 mothers participated in the endline survey. At endline, there was a risk difference of 38·9% (95% CI 32·2–45·6, p<0·001) between the reported prevalence of exclusive breastfeeding in the intervention group and that of the control group.

**Interpretation:**

A multidimensional intervention deliverable at scale in a low-income setting resulted in substantial increases in mothers' optimal breastfeeding knowledge and beliefs and in reported exclusive breastfeeding practices. However, it is possible that the findings might have been influenced by social desirability bias.

**Funding:**

Bill & Melinda Gates Foundation, London School of Hygiene & Tropical Medicine.

## Introduction

The many benefits of exclusive breastfeeding on child mortality, health, and development have been well documented.^1^ As such, WHO recommends exclusive breastfeeding as the optimal form of feeding for infants aged up to 6 months, with continued breastfeeding alongside complementary feeding for infants aged between 6 months and 2 years or older.^2^

Interventions to promote breastfeeding are most successful when they take a complex multidimensional approach, targeting a broad range of domains, including policy environment, social attitudes and norms among both mothers and their wider community, and supportive health-care services.^3^, ^4^, ^5^ A systematic review and meta-analysis^6^ published in 2018, found that several strategies can improve the effectiveness of infant feeding interventions: the use of a multidimensional intervention taking place in both facility and community settings, involvement of health providers, use of a precise protocol for provider training, and use of interventions that take place over a period that includes pregnancy and postpartum. Another systematic review^7^ found that interventions delivered in a combination of settings, and interventions that were provided concurrently at the facility and in the community, achieved the greatest improvement in exclusive breastfeeding and other breastfeeding outcomes.

One such multidimensional approach, the Alive & Thrive initiative, combines different programme components, in both community and facility settings, to improve infant feeding in low-income regions. Alive & Thrive's theory of change describes a framework in which interpersonal communication, community mobilisation, advocacy, mass communication activities, and strategic use of data act in synergy to improve knowledge, beliefs, skills, and an enabling environment within the community to ultimately improve breastfeeding and complementary feeding practices and health outcomes.^8^ The interpersonal communication component of Alive & Thrive consists of enhanced training of existing cadres—both health workers within government facilities and community health workers—and is designed to fit within existing structures rather than as a parallel intervention. Alive & Thrive is designed to be delivered at scale and reach large numbers of mothers and infants to have an effect at the regional and national levels. The initiative also targets the broader community in addition to mothers themselves. As such, its underlying framework is different from another breastfeeding intervention^9^ previously trialled in Burkina Faso, which focused on intensive repeat contacts at the individual level. In Burkina Faso, Alive & Thrive originally aimed to increase exclusive breastfeeding prevalence in areas reached by the initiative to at least 50% of infants younger than 6 months (from the national prevalence of 25% reported in the 2010 Demographic and Health Survey)^10^ over 3 years, starting from 2014. Nationally, the programme hoped to reach half of the population; in the region of Boucle du Mouhoun, where the study took place, Alive & Thrive aimed to reach 80% of the target groups.

**Research in context**

**Evidence before this study**

The benefits of exclusive breastfeeding on infant and child health and mortality have been well documented. Several published systematic reviews have concluded that multidimensional interventions taking place over a period including both pregnancy and the post-partum period are most likely to be effective in increasing exclusive breastfeeding practices. However, few such interventions have been assessed at scale with a rigorous design in an African setting.

**Added value of this study**

This study describes a cluster-randomised controlled trial assessing the Alive & Thrive intervention package in Burkina Faso. The interventions assessed within the package included provision of training, supportive supervision, job aids, and communications materials to both health workers working at the primary care level (local health centres) and the volunteer community health worker cadre who operate in villages. The focus of the intervention was on improving the quality of breastfeeding counselling provided to pregnant and breastfeeding mothers during visits to health centres and home visits. These health system interventions were combined with community mobilisation activities, which targeted husbands, grandmothers, and the wider community to create an enabling environment that would support pregnant women and mothers to adopt optimal breastfeeding feeding practices. We showed that multidimensional interventions, such as Alive & Thrive, can be successfully delivered in the context of Burkina Faso and that Alive & Thrive resulted in substantial increases in optimal breastfeeding knowledge and beliefs and in reported breastfeeding practices.

**Implications of all the available evidence**

Multidimensional breastfeeding interventions delivered at scale can improve infant feeding knowledge, beliefs, and practices in low-income settings.

Few studies combine at-scale programmes, such as Alive & Thrive, with randomised assessment methods.^11^ We did a randomised impact assessment of Alive & Thrive with the aim of assessing the effect of Alive & Thrive's locally delivered components (namely, interpersonal communication and community mobilisation activities) on the prevalence of exclusive breastfeeding among infants aged 6 months or younger and on other secondary outcomes in infants aged 12 months or younger, in Boucle du Mouhoun, Burkina Faso.

## Methods

### Study design and participants

Our study was a cluster-randomised controlled trial done in Boucle du Mouhoun, Burkina Faso. We selected a clustered design because some components of the intervention were delivered at the community level. We used data collected with two cross-sectional surveys: a baseline survey before the start of implementation (June and July, 2015) and an endline survey done in June and July, 2017.

Burkina Faso is a low-income setting with high infant mortality: an early neonatal mortality of 20 deaths per 1000 livebirths, a late neonatal mortality of nine deaths per 1000 livebirths, and a postneonatal mortality of 32 deaths per 1000 livebirths.^12^ Boucle du Mouhoun is a region in the northwest of the country with a population of 1·4 million.^13^ Here, 60% of the population fall below the national poverty line of annual consumption of 154 000 CFA francs (about US$270).^13^ Infant feeding practices in our baseline survey before the intervention have been reported elsewhere.^14^ In brief, we found that in Boucle du Mouhoun, 30% of infants younger than 6 months were reported to have been exclusively breastfed on the day before the interview and giving infants water or other liquids before they were 6 months old was a strong social norm. Very few mothers (9%) initiated breastfeeding within 1 h of delivery, and complementary feeding indicators among older infants were also poor. Three quarters of mothers reported giving colostrum after birth, and 85% reported that they gave no prelacteal feeds.

Women were eligible for inclusion in the baseline and endline surveys if they were residing in a village selected for the study (had been members of households identified during household listing for at least 6 months or had the intention to stay there), had a livebirth in the 12 months preceding either of the two surveys and the infant was still alive and living with them, and gave informed consent to participate. Informed consent was obtained using a three-step approach. First, representatives of the communities involved were asked to identify whether there were any concerns relating to the study. Second, women invited to participate were consulted as to whether the study team should request their husband's permission before participation. Finally, individual informed consent was sought from mothers with an information sheet and consent form. Consent for data collection was sought after randomisation, before the interview.

Ethical approval for this study was granted by the National Health Ethics Committee of the Ministry of Health of Burkina Faso (2015–5-061), the institutional review board of Centre MURAZ (2015–017) and the London School of Hygiene & Tropical Medicine (9066). The detailed study protocol is available online.

### Randomisation and masking

A cluster was defined as a rural commune. Boucle du Mouhoun has 41 rural communes and six urban communes;^15^ we excluded the six urban communes because parts of the intervention (specifically the community mobilisation activities) were implemented only in villages by design. We stratified randomisation by province to try to balance potential co-interventions and confounders, such as those that might be associated with ethnic groups. Therefore, 20 clusters were allocated to the intervention arm and 21 clusters were allocated to the control arm by use of computer generated pseudo-random numbers (Stata, version 14.0). Randomisation was done by SC. It was not possible to mask the intervention to the implementing organisations. The data collection team was not told which communes were in the intervention group or which were in the control group. However, this trial should be considered unblinded because of the nature of the intervention.

After randomisation, but before the start of baseline data collection, we learned of a similar intervention on infant and young child feeding taking place in four communes (two in the intervention and two in the control group). The decision was taken to exclude these four communes from the study because of co-intervention, leaving 18 clusters in the intervention group and 19 in the control group in the final design of the study (appendix).

### Procedures

Each survey was an independently selected representative sample of the target population. Participants were sampled by use of a two-stage approach. First, within each cluster, we randomly selected three villages with probability proportional to size, with use of the most recent census (2006) at the time as a sampling frame. Control villages close to the boundaries of intervention communes were excluded from the sampling frame to reduce the risk of contamination. We then did a census of each selected village to identify all eligible mothers within the village. 20 mother–infant pairs (comprising ten infants younger than 6 months and ten aged 6 to 11 months) were sampled per village by stratified simple random sampling.

Data were collected with a structured questionnaire, administered with use of a Trimble Juno SB Personal Digital Assistant (Trimble, Sunnyvale, CA, USA). The baseline and endline questionnaires are available online. Quality assurance mechanisms included the Personal Digital Assistant programme, observed interviews, and checks for consistency and implausible values; interviewers were instructed to return to the household where inconsistencies were identified.

Only interpersonal communication and community mobilisation activities were randomly assigned, and hence assessed, in this study. Other components of the Alive & Thrive's framework not assessed in this study included advocacy, which occurred at the national policy level, and a mass communication radio campaign, which did not air in Boucle du Mouhoun. Alive & Thrive partnered with the government and with the non-governmental organisations Western University Service of Canada, to deliver interpersonal communication activities, and Mwangaza Action, to deliver community mobilisation activities.

The main messages of the Alive & Thrive intervention, delivered through both interpersonal communication and community mobilisation activities were the following: to place the baby to the breast within the first hour of birth, to give colostrum, to not give water, tisanes, or other liquids, and to breastfeed exclusively for 6 months.

The purpose of the interpersonal communication activities was to increase mothers' knowledge about optimal breastfeeding practices and their benefits, to increase mothers' expertise in breastfeeding, and to improve mothers' perceptions about social norms relating to breastfeeding. These activities were delivered by existing structures within the public health system; the intervention primarily consisted of training sessions for staff specific to infant and young child feeding and enhanced supervision and monitoring structures. Communication materials, such as posters, leaflets, and counselling cares, were also developed and shared. Interpersonal communication activities were delivered by two cadres: government health workers during individual consultations for antenatal, delivery, and postnatal care and during women's group discussions held at the local health centre; and community health volunteers during home visits that targeted pregnant and postnatal women who, during a visit to a health centre, expressed concerns or mentioned experiencing difficulties with breastfeeding. Government health facilities and community health volunteers were also present in the control group, as part of the standard public health system, but did not benefit from any additional training, supervision, or community mobilisation activities of Alive & Thrive.

Both government health workers and community health volunteers in the intervention group were supported by enhanced training in infant feeding to improve their ability to support mothers and provide timely information, in line with government guidelines. These trainings took place in May, 2016, with additional training regularly provided for new staff. By June, 2017, Alive & Thrive had trained a total of 1226 community health volunteers and 381 government health workers, in 93 local health centres in Boucle du Mouhoun. Additionally, Alive & Thrive implemented a system of supportive supervision to improve quality of interpersonal communication and equipped all local health centres in the intervention group with communication tools, including counselling cards, posters, mini-posters, and leaflets with short messages on breastfeeding for mothers to take home.

Home visits are part of the routine activities of community health volunteers in Burkina Faso, to support health promotion in the community. However, volunteers are expected to do many other tasks, and home visits are a small component of their role. There are two community health volunteers per village and, as part of the intervention, they agreed to each carry out four home visits per month (two visits to pregnant and two to breastfeeding mothers); however, we did not expect that they would be able to provide high coverage. Community health volunteers were instructed to prioritise mothers who had been identified by government health workers as needing additional support, such as those who had expressed concerns about breastfeeding during a visit to a health facility; this decision was taken to prioritise resources and ensure that the intervention was deliverable at scale, because of the volunteer nature of this cadre. Use of antenatal care is very high in Boucle du Mouhoun (94% of pregnant women),^10^ thus most women were in contact with the health system.

The purpose of the community mobilisation activities was to raise awareness of the benefits of breastfeeding primarily among partners, mothers-in-law, and grandmothers and to increase the support that they and the community provide to breastfeeding mothers. Pregnant women and breastfeeding mothers were a secondary target of the community mobilisation activities, which consisted of community events and facilitated group discussions in public places in the villages to promote recommended breastfeeding practices. Some community health volunteers also received requests to support wives or daughters-in-law during their interactions with fathers and grandmothers at these events. Community mobilisation activities took place from December, 2015 to July, 2017, done by 40 trained community workers and five supervisors, and reached 399 villages during this period, with each community worker being responsible for nine or ten villages. At least one meeting with each primary target per village per month was done.

### Outcomes

The primary outcome of the trial was prevalence of *exclusive breastfeeding*, defined as the proportion of infants younger than 6 months reported to have received only breastmilk during the day and night before the survey.^16^ In line with the WHO definition, infants could receive expressed breastmilk, modern medicines (including vitamin and mineral syrups), and oral rehydration solutions, and still be considered as exclusively breastfed. We did not ask mothers directly if the infant was exclusively breastfed; this variable was generated at the analysis stage on the basis of a list of 29 foods and liquid items reported as consumed (or not).

Secondary outcomes of the trial were the following: prevalence of early initiation of breastfeeding, defined as the proportion of currently living infants aged 11 months or younger who were placed on the breast within 1 h of birth; percentage of currently living infants aged 11 months or younger who were given colostrum; percentage of currently living infants aged 11 months or younger who did not receive any prelacteal feedings, defined as any foods or liquids other than breastmilk given within the first 3 days of life; and continued breastfeeding as a proportion of infants aged 6–11 months who were breastfed during the day and night before the survey.^16^ All indicators of the knowledge and opinions of the mother relating to infant feeding are provided in this study as the percentage of currently living infants aged 11 months or younger. Regarding data on exposure to components similar to those within the Alive & Thrive intervention, we asked mothers if they had received a particular type of intervention, but we did not expect or ask mothers to report on who had organised that component. Other secondary outcomes relating to complementary feeding that was not explicitly targeted by the Alive & Thrive initiative (ie, introduction of semi-solid, solid, or soft foods; minimum acceptable diet; and dietary diversity) will be reported elsewhere (Sarrasat S and colleagues, unpublished).

### Statistical analysis

Sample size was calculated with the Hayes and Moulton method.^17^ During the development of the protocol, we assumed prevalence of exclusive breastfeeding in the control group to be 30%, on the basis of the region-specific prevalence for Boucle du Mouhoun from the demographic health survey.^18^ Our original design had 20 clusters per group, each recruiting 30 mother–infant pairs with infants younger than 6 months, and an additional 30 mother–infant pairs, with infants aged 6–11 months, per cluster (equal sized clusters), giving a total sample size of 2400 mother–infant pairs (1200 mother–infant pairs with infants younger than 6 months). We estimated this would provide at least 90% power for us to detect an absolute difference in exclusive breastfeeding prevalence among infants younger than 6 months between intervention and control clusters of 50% versus 30%, assuming a coefficient of variation (*k*) of 0·4 on the basis of the coefficient of variation of 0·33 found in the intervention group of a previous trial of exclusive breastfeeding in Burkina Faso,^9^ and an α of 5%. After four communes were excluded, the sample size was 2160 mother–infant pairs, which would still allow a difference of 50% versus 30% to be detected with a power of at least 87%.

Our analysis plan is described in the study protocol. Analyses were done with Stata (version 14.0). Baseline data were inspected for balance between the trial groups. Our main analysis was based on a logistic regression model fitted to the individual-level data, with robust standard errors that allowed for intragroup correlation; the margins command was used to obtain an estimate of the risk of exclusive breastfeeding in both groups, and the adjrr command was used to give the adjusted risk difference between the intervention and control groups.^19^ Cluster-level prevalence at baseline was controlled for as a covariate in the models. We also did a difference-in-difference analysis on the cluster-level summary data. Finally, we did an additional supplementary analysis within the intervention group at endline, which was not prespecified, comparing women's report of exposure to different components of the Alive & Thrive initiative with exclusive breastfeeding. The trial is registered with ClinicalTrials.gov, number NCT02435524.

### Role of the funding source

The funders of the study had no role in study design, data collection, data analysis, data interpretation, or writing of the report. The corresponding author had full access to all the data in the study and had final responsibility for the decision to submit for publication.

## Results

Between June 2 and July 28, 2015, 2288 mothers participated in the baseline survey (1173 mothers of infants younger than 6 months and 1115 mothers of infants aged 6–11 months), and between June 12 and July 25, 2017, 2253 mothers participated in the endline survey (1136 mothers of infants younger than 6 months and 1117 mothers of infants aged 6–11 months; figure). Table 1 presents the baseline characteristics of the study population. The trial arms were reasonably balanced regarding the primary and secondary outcomes, as well as key socio-demographic characteristics.

**Figure:**
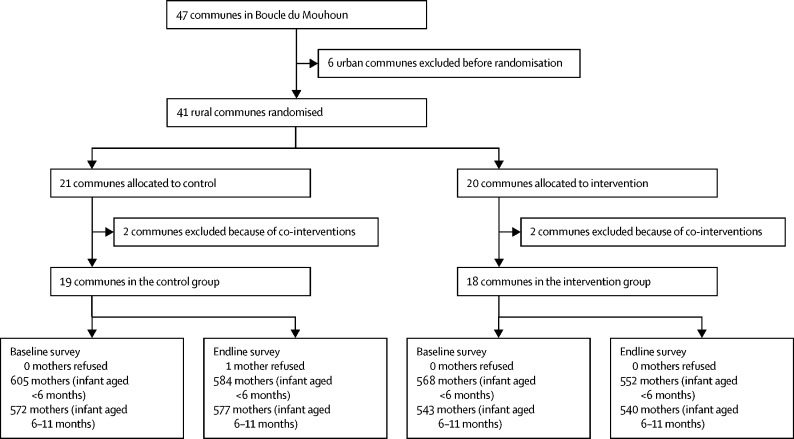
Flow chart of participant recruitment

**Table 1:** Baseline characteristics stratified by trial group

		**Controlgroup**	**Intervention group**
Exclusive breastfeeding (infants aged <6 months)		162 (26·8%)	190 (33·5%)
	Total	605 (100%)	568 (100%)
Continued breastfeeding (infants aged 6–11 months)		572 (100%)	543 (100%)
	Total	572 (100%)	543 (100%)
Early initiation of breastfeeding		118 (10·0%)	96 (8·6%)
Gave colostrum		857 (72·8%)	850 (76·5%)
Received no prelacteal feeds		961 (81·6%)	977 (87·9%)
Age (years)			
	15–24	448 (38·1%)	401 (36·1%)
	25–34	519 (44·1%)	467 (42·0%)
	35–49	210 (17·8%)	243 (21·9%)
	Mean age in years (SD)	27·4 (6·9)	27·9 (7·0)
	Median age in years (IQR)	27 (22–32)	27 (22–33)
Parity (including index birth)			
	1	213 (18·1%)	181 (16·3%)
	2–3	367 (31·2%)	351 (31·6%)
	4–6	427 (36·3%)	378 (34·0%)
	≥7	170 (14·4%)	201 (18·1%)
	Mean parity (95% CI)	3·9 (3·7–4·1)	4·1 (3·9–4·3)
	Median parity (IQR)	4 (2–5)	4 (2–6)
Ethnicity			
	Bobo	137 (11·6%)	141 (12·7%)
	Bwaba	129 (11·0%)	160 (14·4%)
	Dafing	269 (22·9%)	269 (24·2%)
	Mossi	272 (23·1%)	194 (17·5%)
	Peulh	74 (6·3%)	80 (7·2%)
	Samo	109 (9·3%)	181 (16·3%)
	Other	187 (15·9%)	86 (7·7%)
Religion			
	Animist	117 (9·9%)	95 (8·6%)
	Catholic	212 (18·0%)	229 (20·6%)
	Muslim	753 (64·0%)	674 (60·7%)
	Protestant	77 (6·5%)	101 (9·1%)
	Other or no religion	18 (1·5%)	12 (1·1%)
Education			
	No formal schooling	888 (75·4%)	830 (74·7%)
	Primary education only	206 (17·5%)	200 (18·0%)
	Secondary education or higher	83 (7·1%)	81 (7·3%)
Marital status			
	Married or cohabiting: monogamous	734 (62·4%)	701 (63·1%)
	Married or cohabiting: polygamous	420 (35·7%)	380 (34·2%)
	Divorced or separated, widowed, or single	23 (2·0%)	30 (2·7%)
Relative wealth quintile			
	Poorest	216 (18·4%)	237 (21·3%)
	Poorer	242 (20·6%)	213 (19·2%)
	Middle	222 (18·9%)	232 (20·9%)
	Richer	234 (19·9%)	222 (20·0%)
	Richest	252 (21·4%)	203 (18·3%)
Total		1177 (100%)	1111 (100%)

Data are n (%), unless otherwise specified.

Our findings for the primary outcome showed a difference of 38·9% (95% CI 32·2–45·6, p<0·001) in the reported prevalence of exclusive breastfeeding between the control and intervention groups at endline (table 2). A breakdown of infant feeding by food type is provided in the appendix. At endline, the *k* value was 0·63 overall, 0·98 in the control group, and 0·29 in the intervention group. The findings stratified by province are provided in the appendix. The reported prevalence of exclusive breastfeeding increased substantially in both the intervention and control groups.

**Table 2:** Prevalence of reported exclusive breastfeeding and secondary breastfeeding outcomes at endline, as calculated with a generalised linear model on individual-level data

	**Baseline**	**Endline**			
		Control group	Intervention group	Risk difference	p value
Exclusive breastfeeding (infants aged <6 months)	1173 (30·0%, 23·4 to 36·6)	584 (53·6%, 47·7 to 59·5)	552 (92·5%, 89·3 to 95·7)	38·9% (32·2 to 45·6)	<0·0001
Early initiation of breastfeeding (infants aged <12 months)	2288 (9·4%, 7·4 to 11·3)	1161 (14·3%, 9·4 to 19·2)	1092 (37·0%, 30·4 to 43·6)	22·7% (14·6 to 30·8)	<0·0001
Gave colostrum (infants aged <12 months)	2288 (74·6, 71·1 to 78·1)	1161 (75·6%, 70·9 to 80·3)	1092 (95·6%, 93·2 to 98·1)	20·0% (14·7 to 25·4)	<0·0001
Received no prelacteal feed (infants aged <12 months)	2288 (84·7%, 80·5 to 88·9)	1161 (90·3%, 98·5 to 99·8)	1092 (99·2%, 98·5 to 99·8)	8·8% (5·8 to 11·9)	<0·0001
Continued breastfeeding (infants aged 6–11 months)	1115 (100%)	577 (99·8%, 99·4 to 100)	540 (100%)	0·2% (−0·2 to 0·5)	0·333

Data are n (%, 95% CI), unless otherwise specified.

Other secondary breastfeeding outcomes are also presented in table 2. In the intervention group, we found an increase in the prevalences of mothers reporting early initiation of breastfeeding (22·7% difference), giving colostrum (20·0% difference), and no prelacteal feeds (8·8% difference) compared with those in the control group. We found continued breastfeeding among almost all older infants in both study groups. A breakdown by age group is provided in the appendix.

Mothers in the intervention group had improved knowledge on the optimal timing of breastfeeding initiation and duration of exclusive breastfeeding (table 3). We observed higher prevalences of mothers responding that an infant should be placed on the breast within 1 h of delivery and mothers saying that they should exclusively breastfeed for 6 months in the intervention group compared with those in the control group (table 3).

**Table 3:** Prevalence of correct knowledge relating to optimal breastfeeding practices at endline, as calculated with a generalised linear model on individual-level data

	**Baseline (n=2288)**	**Endline**			
		Control group (n=1161)	Intervention group (n=1092)	Risk difference	p value
A mother should start breastfeeding during the first hour after delivery	38·8% (35·3–42·5)	50·3% (43·0–57·6)	76·9% (72·9–80·9)	26·6% (18·4–34·7)	<0·001
A mother should breastfeed exclusively for the first 6 months	51·0% (46·2–55·9)	57·2% (50·1–64·2)	80·3% (76·1–84·6)	23·1% (14·9–31·3)	<0·001
A mother should start to give water or other liquids to her infant after age 6 months	Not collected at baseline	53·9% (45·5–62·3)*	89·9% (85·9–93·9)*	36·0% (27·5–44·5)*	<0·001*

Data are % (95% CI).

* Endline survey analysis did not adjust for baseline rates.

The extent to which mothers agreed with a series of statements relating to breastfeeding is described in table 4. Mothers generally saw breastfeeding as a positive thing and overwhelmingly agreed it was good for the health of infant and mother across the intervention and control groups. Mothers in the intervention group were more likely to believe that a breastfed baby would have less diarrhoea, to disagree that colostrum was not good for the baby's health, and to disagree that tisanes protected the baby's health (table 4). Importantly, the proportion of mothers who thought that a baby needed to drink water in addition to breastmilk was reduced by half in the intervention group compared with that in the control group.

**Table 4:** Mothers' opinions relating to breastfeeding practices at endline, as calculated with a generalised linear model on individual-level data

	**Baseline (n=2288)**	**Endline**			
		Control group (n=1161)	Intervention group (n=1092)	Risk difference	p value
“Breastfeeding is a good thing for the health of the baby”	99·0% (98·3 to 99·6)	99·5% (99·1 to 99·8)	99·5% (98·8 to 100)	0·0% (−0·8 to 0·9)	0·904
“Breastfeeding is a good thing for the health of the mother”	88·4% (83·8 to 92·9)	97·0% (96·0 to 97·9)	96·8% (95·6 to 98·1)	−0·1% (−1·7 to 1·4)	0·876
“If a mother breastfeeds, the baby will have less diarrhoea”	76·5% (73·6 to 79·5)	87·7% (84·9 to 90·5)	93·6% (92·0 to 95·1)	5·9% (2·7 to 9·1)	<0·001
“To give colostrum to a baby is not a good thing for their health”	45·5% (41·4 to 49·6)	42·4% (38·3 to 46·5)	25·9% (20·5 to 31·3)	−16·5% (−23·3 to −9·8)	<0·001
“Cow's milk is more nutritious for babies than breastmilk”	8·1% (6·6 to 9·6)	9·6% (6·9 to 12·4)	8·7% (6·6 to 10·7)	−1·0% (−4·5 to 2·5)	0·587
“If a mother breastfeeds, the baby will have fewer illnesses”	84·4% (81·9 to 86·9)	91·3% (89·1 to 93·6)	94·5% (92·9 to 96·0)	3·1% (0·4 to 5·8)	0·025
“A baby needs to drink water in addition to breastmilk”	74·4% (70·7 to 78·0)	69·2% (63·8 to 74·6)	33·8% (25·7 to 41·9)	−35·4% (−45·2 to −25·7)	<0·001
“Tisanes* and infusions protect a baby's health”	65·1% (59·9 to 70·0)	65·0% (60·6 to 69·5)	29·0% (24·2 to 33·8)	−36·1% (−43·0 to −29·1)	<0·001
“While a mother is exclusively breastfeeding her baby, she can avoid pregnancy”	29·1% (24·1 to 34·1)	39·2% (35·7 to 42·6)	43·8% (39·9 to 47·8)	4·7% (−0·6 to 9·9)	0·079

Data are % (95% CI) of mothers who agree with the given statements.

* A tisane is a herbal tea used locally.

The difference-in-difference approach resulted in broadly similar conclusions, with the exception of prelacteal feeds: we found no significant difference in the proportion of mothers reporting no prelacteal feeds, which was very high in both groups, with this approach (appendix).

The intervention was successfully delivered at scale: 1050 (96%) of 1092 mothers in the intervention group reported exposure to at least one component at endline, with a mean of 6·6 (SD 4·8) exposures during pregnancy and the post-partum period (appendix). The associations between exposure to individual intervention components and improved outcomes were consistently in the expected direction, although the risk differences for individual components were generally modest (appendix).

## Discussion

The locally-delivered components of the Alive & Thrive initiative improved the knowledge, attitudes, and mothers' reporting of exclusive breastfeeding practices by the time of the endline survey. Although the findings of our study are overall positive, it should be noted that harmful norms surrounding infant feeding, such as feeding water and infusions to very young infants, persist in Burkina Faso. We also observed substantial changes in the control group between baseline and endline surveys, which are possibly due to co-intervention, contamination, and secular trends.

Our findings are similar to another breastfeeding intervention in Burkina Faso, the PROMISE-EBF trial,^9^ which found that peer-counselling visits increased the reported exclusive breastfeeding prevalence at 24 weeks, from 22% to 73%. The Alive & Thrive initiative also included home visits, although these were done by trained community health workers rather than by peer support, alongside the other components of the intervention. The Alive & Thrive framework was designed to enhance the training and supervision of existing resources and structures in the health system, in combination with community mobilisation and advocacy to target norms. The target audience of the Alive & Thrive initiative is broader than that of the PROMISE-EBF trial, involving the mother's family and the broader community in addition to the mother herself. A systematic review^20^ published in 2017, found that interventions delivered concurrently in a combination of facility, home, and community settings showed the largest improvements in breastfeeding outcomes in low-income and middle-income settings. The Alive & Thrive framework has been assessed in other settings, such as Bangladesh, Ethiopia, and Vietnam, which have similarly shown that at-scale interventions combining interpersonal counselling and community mobilisation components can improve breastfeeding practices.^21^, ^22^ In Burkina Faso, the Alive & Thrive initiative was not without its challenges, such as the mobility of health workers and workload concerns from health workers completing the monitoring tools. Adaptations were made to increase human resources, increase supervision, and modify training schedules, and monitoring tools were implemented to address these concerns. Ultimately, sustainability studies are needed to provide guidance on which solutions are the most cost-effective and which ones might vary by setting.

It is encouraging that secular trends, or changes over time, in the control group were also positive. Our baseline survey in 2015, found an exclusive breastfeeding prevalence among mothers of 30% within Boucle du Mouhoun,^14^ a survey by the Ministry of Health found a prevalence of 43% in 2016,^23^ and the prevalence in our control group in 2017 was 51%. Because of the randomised design of our study, we do not believe that secular trends explain the difference between the intervention and control groups. Contamination could have occurred because of staff turnover of health workers within the local health centres and inter-village events, although we believe the effect of inter-village events was minor because we excluded villages in the control group that were close to the intervention area. Nonetheless, it is possible that the energy of the Alive & Thrive message and the commitment of our partners to the importance of exclusive breastfeeding spread at the district and regional level, resulting in improved commitment to existing government guidelines even in the control group. The Alive & Thrive framework also includes national-level policy advocacy, which was not possible to randomise and thus was not included in this study, but might have filtered down.

In our study, exclusive breastfeeding was measured by use of the mother's report of her infant's diet the day and night preceding the survey. Mothers in the intervention group were exposed to many messages promoting positive infant feeding practices during the 18 months preceding the endline survey, and it is possible that their responses might have been influenced by social desirability bias. A biological validation study comparing mothers' reports with the deuterium oxide turnover technique was done in the study area in 2016. Compared with exclusive breastfeeding measured with the deuterium oxide turnover technique, mothers' reports overestimated exclusive breastfeeding in both groups of that study. This overreporting was greater in the intervention group than in the control group (Diallo and colleagues, unpublished). In 2016, when this validation study took place, the intervention had started, but had not been completely rolled out. It is plausible that changes in knowledge and social desirability would precede actual behaviour changes; it is also possible that other people, such as the grandmother or mother-in-law, might have introduced non-breastmilk liquids without the mother's knowledge. It is clear from our data that mothers' knowledge improved and exclusive breastfeeding was viewed more positively as a result of the intervention, a necessary step on the pathway to changing behaviour. Nonetheless, we know from other public health challenges, such as smoking cessation interventions,^24^ that there can be a considerable lag between information becoming widely known and behaviour change occurring.

The key strength of our study was the randomised design, which increased our confidence in attributing the observed changes in outcomes to the interventions. We had a large sample size, in terms of both individuals and clusters, resulting in a good balance between the study groups regarding our outcomes and key sociodemographic confounders at baseline. Our study also had several limitations: data were based on the mother's self-reports, and it is possible that other members of the household and caregivers might also have fed the infant food or liquids with or without the mother's knowledge. We asked about individual food components to measure exclusive breastfeeding to reduce the risk of courtesy bias (social or cultural pressure that a responder feels to give what they believe to be the right answer). We collected data on live infants, which might have biased secondary outcomes in which data were based on recall, but it did not influence the primary outcome of exclusive breastfeeding prevalence. Our data were collected over an approximately 6-week period during June and July, 2015 and 2017: because of seasonality, we might have obtained different absolute results than those if the survey had been done at another time of year. However, this should not influence the conclusions relating to the effectiveness of the Alive & Thrive intervention because the data were collected at a comparable timepoint. We were unable to investigate any therapist effect.

Overall, a multidimensional intervention deliverable at scale in a low-income setting resulted in substantial increases in optimal breastfeeding knowledge and beliefs and significant increases in reported breastfeeding practices. Additional studies are needed to assess the intervention's effect on infant and child health and development outcomes, as well as the cost-effectiveness of the intervention. Although Alive & Thrive resulted in important improvements in knowledge and positive attitudes, further improvements are needed because harmful norms, such as giving young infant's water in addition to breastmilk, are still pervasive in Burkina Faso, in common with elsewhere in west Africa.

For the **study protocol** see https://doi.org/10.17037/DATA.280

For the **baseline questionnnaire** see https://doi.org/10.17037/DATA.173

For the **endline questionnnaire** see https://doi.org/10.17037/DATA.280
