# Supplementary material for: The effect of the Alive & Thrive initiative on exclusive breastfeeding in rural Burkina Faso: a repeated cross-sectional cluster randomised controlled trial
Source: Lancet Glob Health. 2019 Feb 14;7(3):e357–65. doi: 10.1016/S2214-109X(18)30494-7 (PMC6379822; doi:10.1016/S2214-109X(18)30494-7)
Supplement: Supplementary appendix [file mmc1.pdf]

THE LANCET
Global Health

Supplementary appendix

This appendix formed part of the original submission and has been peer reviewed. We post it as supplied by the authors.

Supplement to: Cresswell JA, Ganaba R, Sarrassat S, et al. The effect of the Alive & Thrive initiative on exclusive breastfeeding in rural Burkina Faso: a repeated cross-sectional cluster randomised controlled trial. *Lancet Glob Health* 2019; **7:** e357–65.

Supplementary Web Appendix

Jenny A Cresswell, Rasmané Ganaba, Sophie Sarrassat, Henri Somé, Abdoulaye Hama Diallo, Simon Cousens, Veronique Filippi. "A repeated cross-sectional cluster randomised controlled trial to investigate the effect of the Alive & Thrive initiative on exclusive breastfeeding in rural Burkina Faso" Lancet Global Health

Figure S1: Map of communes randomised

Pink cross-hatch = control commune; green stripes = intervention commune; solid grey = excluded commune

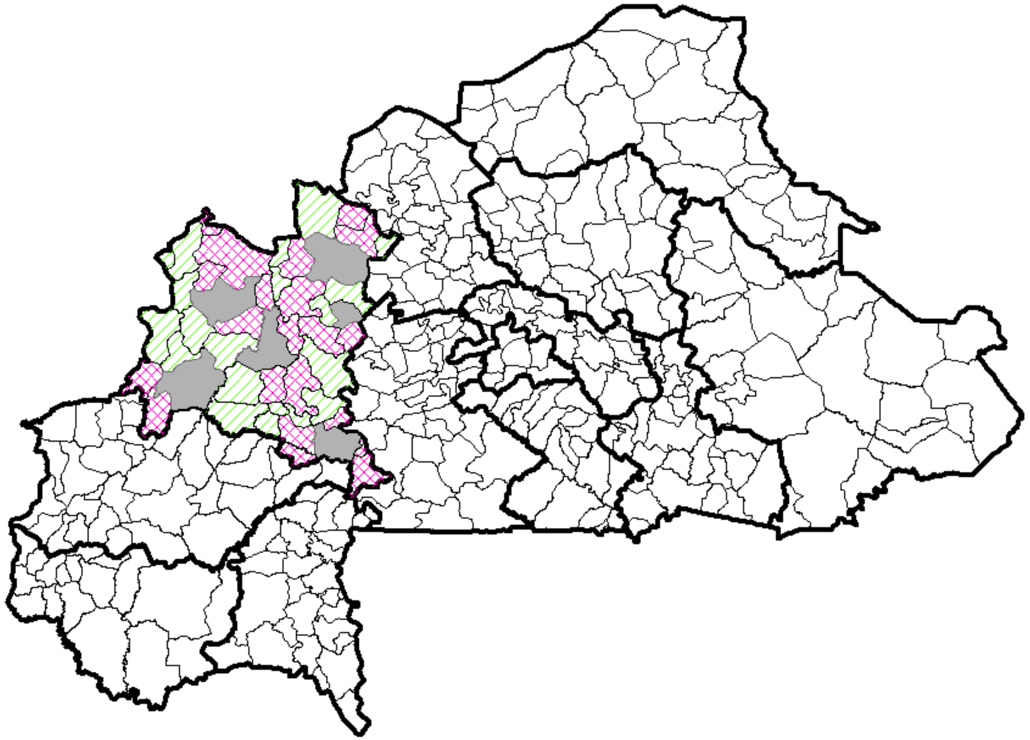

Figure S2: Risk difference in exclusive breastfeeding, stratified by province

Note. 20% indicates the hypothesised effect size a priori

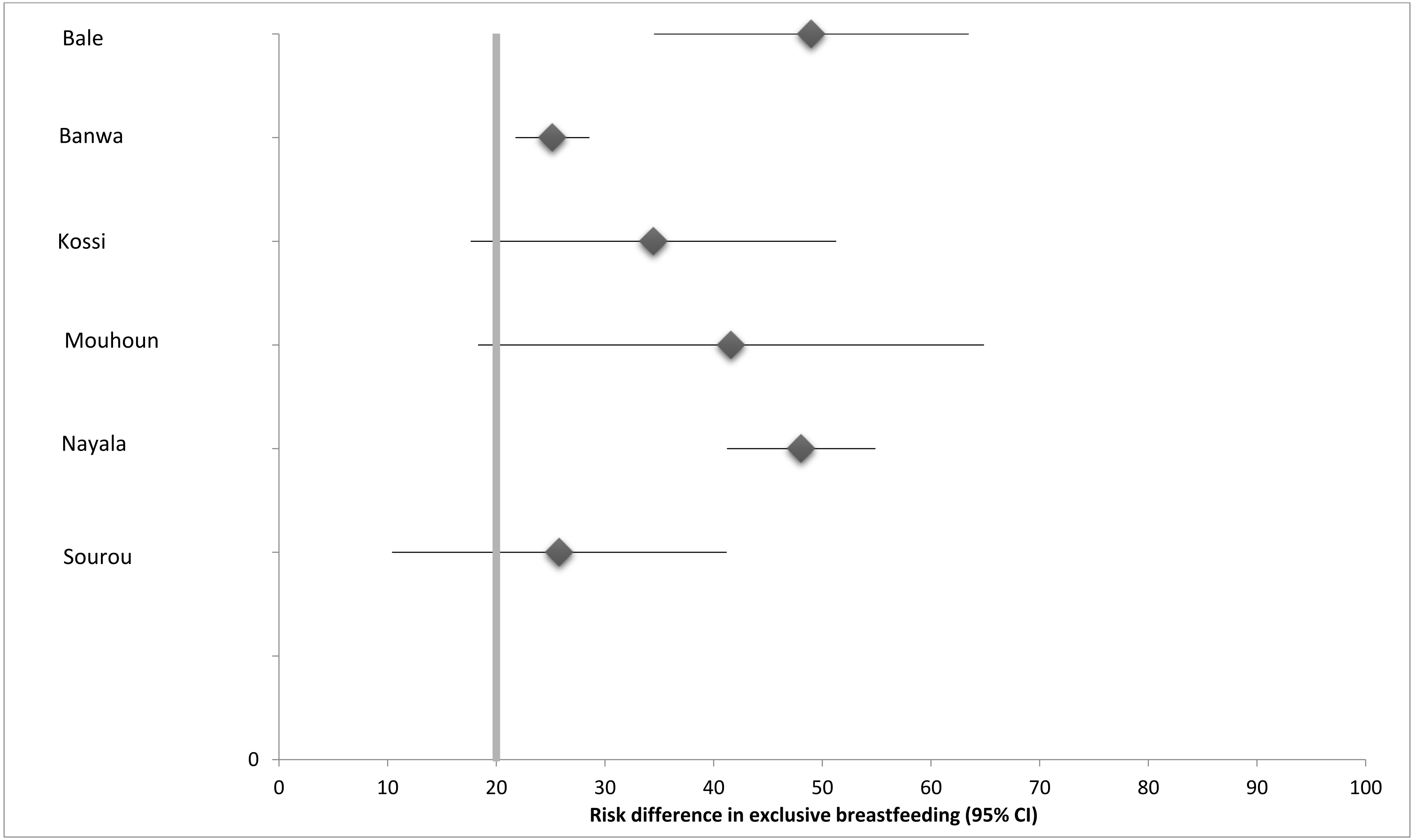

Table S1A: Food and liquid consumption in the day and night preceding the survey among infants <6 months at endline; breakdown of exclusive breastfeeding

	**Control Arm N (%)**	**Intervention Arm N (%)**
Pure water	254 (43.5%)	31 (5.6%)
Sugary or salty water	7 (1.2%)	1 (0.2%)
Coffee or tea	11 (1.9%)	2 (0.4%)
Infusion	123 (21.6%)	6 (1.1%)
Fruit juice	2 (0.3%)	0
Dolo (locally-brewed alcoholic drink) or other alcohol	1 (0.2%)	2 (0.4%)
Cow or goat's milk	2 (0.3%)	1 (0.2%)
Formula/powdered milk substitute	3 (0.5%)	1 (0.2%)
Other liquid	0	0
Yoghurt; curds	0	1 (0.2%)
Soup	3 (0.5%)	0
Bouille (thin gruel/porridge)	10 (1.7%)	3 (0.5%)
Cereal-based dish such as rice, pasta, millet or tô	2 (0.3%)	2 (0.4%)
Black-eyed peas	0	0
Yellow/orange vegetables such as carrot or squash	0	0
Tuber-based dish such as potato, yam, or cassava	0	0
Dark green leaves such as spinach	2 (0.3%)	2 (0.4%)
Other vegetables	0	0
Meat or poultry	0	0
Offal	0	0
Eggs	0	0
Fresh or dried fish	1 (0.2%)	0
Mayflies, caterpillars, crickets	0	0
Palm oil	0	0
Mango or papaya	0	0
Other fruit	0	1 (0.2%)
Peanuts or cashew nuts	0	0
Snacks such as cakes, biscuits or crisps	2 (0.4%)	1 (0.2%)
Other semi-solid or solid food	0	0
None of the above (exclusively breastfed)	299 (51.2%)	515 (93.3%)
**Total**	**584**	**552**

Table S1B: Prevalence of secondary breastfeeding outcomes at endline, stratified by age group

	Infant age (months)	Control Arm		Intervention Arm	
		N	% (95% CI)	N	% (95% CI)
Early initiation of breastfeeding	0-5	584	15.1% (9.3%, 20.9%)	552	39.1% (32.4%, 45.9%)
	6-11	577	13.7% (7.7%, 19.7%)	540	34.4% (25.4%, 43.4%)
Gave colostrum	0-5	584	75.9% (68.2%, 83.5%)	552	95.3% (92.0%, 98.5%)
	6-11	577	72.8% (65.7%, 79.9%)	540	96.7% (94.5%, 98.8%)
Received no prelacteal feeds	0-5	584	90.3% (85.1%, 95.3%)	552	99.1% (98.2%, 100%)
	6-11	577	88.6% (84.1%, 93.1%)	540	99.4% (98.8%, 100%)

Table S2: Prevalence of reported exclusive breastfeeding and secondary breastfeeding outcomes at endline, as calculated using a difference-in-difference model on cluster-level data

Outcome	Survey	Trial Arm	Number of Clusters	Cluster Prevalence % (95% CI)	p-value
Exclusive breastfeeding	Baseline	Control Arm	19	26.9%	
		Intervention Arm	18	33.3%	
		Difference	-	6.4%	
	Endline	Control Arm	19	51.2% (42.2%, 60.1%)	<0.001
		Intervention Arm	18	93.2% (89.4%, 97.0%)	
		Difference	-	42.1% (30.7%, 53.4%)	
	Difference-in-Difference		-	35.7% (19.6%, 51.7%)	<0.001
Early initiation of breastfeeding	Baseline	Control Arm	19	10.1%	
		Intervention Arm	18	8.7%	
		Difference	-	-1.4%	
	Endline	Control Arm	19	14.4% (8.8%, 20.0%)	<0.001
		Intervention Arm	18	36.9% (29.8%, 43.9%)	
		Difference	-	22.4% (15.8%, 29.0%)	
	Difference-in-Difference		-	23.8% (14.5%, 33.1%)	<0.001
Gave colostrum	Baseline	Control Arm	19	72.9%	
		Intervention Arm	18	76.5%	
		Difference	-	3.7%	
	Endline	Control Arm	19	74.5% (67.7%, 81.2%)	<0.001
		Intervention Arm	18	96.0% (93.6%, 98.4%)	
		Difference	-	21.5% (14.6%, 28.4%)	
	Difference-in-Difference		-	17.9% (8.1%, 27.6%)	<0.001
Received no prelacteal feeds	Baseline	Control Arm	19	81.8%	
		Intervention Arm	18	87.9%	
		Difference	-	6.1%	
	Endline	Control Arm	19	89.5% (85.2%, 93.8%)	0.003
		Intervention Arm	18	99.3% (98.7%, 99.8%)	
		Difference	-	9.8% (3.5%, 16.2%)	
	Difference-in-Difference		-	3.7% (-5.3%, 12.7%)	0.414
Continued breastfeeding	Baseline	Control Arm	19	100%	
		Intervention Arm	18	100%	
		Difference	-	0%	
	Endline	Control Arm	19	99.8% (99.5%, 100%)	0.173
		Intervention Arm	18	100% (100%, 100%)	
		Difference	-	0.2% (-0.1%, 0.4%)	
	Difference-in-Difference		-	0.2% (-0.2%, 0.5%)	0.334

Table S3: Prevalence of correct knowledge relating to optimal breastfeeding practices at endline, as calculated using a difference-in-difference model on cluster-level data

Outcome	Survey	Trial Arm	Number of Clusters	Cluster Prevalence % (95% CI)	p-value
A mother should initiate breastfeeding during the first hour after delivery	Baseline	Control Arm	19	37.0%	
		Intervention Arm	18	40.6%	
		Difference	-	3.6%	
	Endline	Control Arm	19	50.7% (42.4%, 59.1%)	
		Intervention Arm	18	76.6% (72.0%, 81.1%)	<0.001
		Difference	-	25.8% (17.6%, 34.0%)	
	Difference-in-Difference		-	22.3% (10.7%, 33.8%)	<0.001
A mother should breastfeed exclusively for the first six months	Baseline	Control Arm	19	50.5%	
		Intervention Arm	18	51.7%	
		Difference	-	1.2%	
	Endline	Control Arm	19	57.1% (48.6%, 65.5%)	
		Intervention Arm	18	80.5% (75.9%, 85.0%)	<0.001
		Difference	-	23.4% (14.0%, 32.8%)	
	Difference-in-Difference		-	22.2% (8.9%, 35.5%)	0.001

Table S4: Mother's opinions relating to breastfeeding practices at endline, as calculated using a difference-in-difference model on cluster-level data

Mothers who agree with	Survey	Trial Arm	Number of Clusters	Cluster Prevalence % (95% CI)	p-value
"Breastfeeding is a good thing for the health of the baby"	Baseline	Control Arm	19	98.6%	
		Intervention Arm	18	99.3%	
		Difference		0.6%	
	Endline	Control Arm	19	99.4% (99.0%, 99.8%)	
		Intervention Arm	18	99.5% (98.9%, 100.0%)	0.768
		Difference		0.1% (-0.9%, 1.1%)	
	Difference-in-Difference			-0.5% (-2.0%, 0.9%)	0.492
"Breastfeeding is a good thing for the health of the mother"	Baseline	Control Arm	19	88.8%	
		Intervention Arm	18	88.2%	
		Difference		-0.7%	
	Endline	Control Arm	19	97.0% (96.0%, 98.0%)	
		Intervention Arm	18	96.8% (95.3%, 98.3%)	0.949
		Difference		-0.2% (-6.7%, 6.3%)	
	Difference-in-Difference			0.5% (-8.7%, 9.6%)	0.922
"If a mother breastfeeds, the baby will have less diarrhoea"	Baseline	Control Arm	19	77.9%	
		Intervention Arm	18	75.3%	
		Difference		-2.7%	
	Endline	Control Arm	19	88.0% (84.8%, 91.1%)	
		Intervention Arm	18	93.4% (91.5%, 95.3%)	0.029
		Difference		5.4% (0.6%, 10.3%)	
	Difference-in-Difference			8.1% (1.2%, 15.0%)	0.022
"To give colostrum to a baby is not a good thing for their health"	Baseline	Control Arm	19	44.7%	
		Intervention Arm	18	43.4%	
		Difference		-4.4%	
	Endline	Control Arm	19	42.9% (38.0%, 47.9%)	
		Intervention Arm	18	25.3% (19.4%, 31.2%)	<0.001
		Difference		-17.6% (-25.3%, -10.0%)	
	Difference-in-Difference			-13.3% (-24.1%, -2.4%)	0.017
"Cow's milk is more nutritious for babies than breastmilk"	Baseline	Control Arm	19	9.0%	
		Intervention Arm	18	7.2%	
		Difference		-1.8%	
	Endline	Control Arm	19	9.6% (6.7%, 12.5%)	
		Intervention Arm	18	8.7% (6.4%, 11.0%)	0.581
		Difference		-0.9% (-3.7%, 5.5%)	
	Difference-in-Difference			0.9% (-3.7%, 5.5%)	0.690
"If a mother breastfeeds, the baby will have fewer illnesses"	Baseline	Control Arm	19	84.8%	
		Intervention Arm	18	84.0%	
		Difference		-0.8%	
	Endline	Control Arm	19	91.4% (89.0%, 93.8%)	
		Intervention Arm	18	94.4% (92.6%, 96.2%)	0.146
		Difference		3.0% (-1.1%, 7.1%)	
	Difference-in-Difference			3.8% (-2.0%, 9.6%)	0.192
"A baby needs to drink water in addition to breastmilk"	Baseline	Control Arm	19	73.7%	
		Intervention Arm	18	75.1%	
		Difference		1.4%	
	Endline	Control Arm	19	68.9% (62.5%, 75.4%)	
		Intervention Arm	18	34.0% (25.3%, 42.7%)	<0.001
		Difference		-34.9% (-43.8%, -26.1%)	
	Difference-in-Difference			-36.3% (-48.8%, -23.8%)	<0.001
"Tisanes* and infusions protect a baby's health"	Baseline	Control Arm	19	71.4%	
		Intervention Arm	18	58.8%	
		Difference		-12.6%	
	Endline	Control Arm	19	68.9% (61.5%, 76.3%)	
		Intervention Arm	18	25.0% (20.4%, 29.6%)	<0.001
		Difference		-43.9% (-52.7%, -35.1%)	
	Difference-in-Difference			-31.3% (-43.8%, -18.9%)	<0.001
"Whilst a mother is exclusively breastfeeding her baby, she can avoid pregnancy"	Baseline	Control Arm	19	29.2%	
		Intervention Arm	18	29.2%	
		Difference		-0.0%	
	Endline	Control Arm	19	39.1% (35.8%, 42.3%)	
		Intervention Arm	18	43.8% (37.3%, 50.4%)	0.270
		Difference		4.7% (-3.8%, 13.2%)	
	Difference-in-Difference			4.8% (-7.2%, 16.8%)	0.430

* A tisane is a herbal tea used locally

Table S5: Reported exposure to interventions similar to those in the A&T initiative among mothers with an infant <12 months (N=2,253)

			Control Arm N=1,161		Intervention Arm N=1,092	
			N	%	N	%
At health facility	During pregnancy or delivery	Received advice from a health worker during antenatal care	389	33.5%	799	73.2%
		Health worker assisted with initiation of breastfeeding	408	35.1%	775	71.0%
		Health worker discussed breastfeeding whilst still at the facility after delivery	457	39.4%	789	72.3%
	Postpartum	Health worker asked about any difficulties breastfeeding during 1st postnatal checkup (for mother)	246	21.2%	466	42.7%
		Health worker observed breastfeeding during 1st postnatal checkup (for mother)	175	15.1%	371	34.0%
		Health worker gave advice on good breastfeeding technique during the 1st postnatal checkup (for mother)	262	22.6%	511	46.8%
		Health worker asked about any difficulties breastfeeding during 2nd postnatal checkup (for mother)	108	9.3%	163	14.9%
		Health worker observed breastfeeding during 2nd postnatal checkup (for mother)	71	6.1%	131	12.0%
		Health worker gave advice on good breastfeeding technique during the 2nd postnatal checkup (for mother)	109	9.4%	188	17.2%
		Health worker asked about any difficulties breastfeeding during 1st postnatal checkup (for infant)	44	3.8%	85	7.8%
		Health worker observed breastfeeding during 1st postnatal checkup (for infant)	29	2.5%	70	6.4%
		Health worker gave advice on good breastfeeding technique during the 1st postnatal checkup (for infant)	54	4.7%	118	10.8%
		Health worker asked about any difficulties breastfeeding during 2nd postnatal checkup (for infant)	19	1.6%	46	4.2%
		Health worker observed breastfeeding during 2nd postnatal checkup (for infant)	21	1.8%	32	2.9%
		Health worker gave advice on good breastfeeding technique during the 2nd postnatal checkup (for infant)	35	3.0%	60	5.5%
		Health worker asked about any difficulties breastfeeding during the most recent weighing appointment	150	12.9%	279	25.5%
		Health worker observed breastfeeding during the most recent weighing appointment	112	9.7%	207	19.0%
		Health worker gave advice on good breastfeeding technique during the the most recent weighing appointment	199	17.1%	370	33.9%
		Health worker asked about any difficulties breastfeeding during the most recent vaccination appointment	195	16.8%	414	37.9%
		Health worker observed breastfeeding during the most recent vaccination appointment	150	12.9%	287	26.3%
		Health worker gave advice on good breastfeeding technique during the the most recent vaccination appointment	255	22.0%	524	48.0%
In the community	During pregnancy	Participated in at least one group meeting about breastfeeding in village	93	8.0%	331	30.3%
		ASBC visited home to give advice on breastfeeding	24	2.1%	167	15.3%
		Participated in at least one group meeting about breastfeeding at the CSPS	171	14.7%	459	42.0%
	Postpartum	Participated in at least one group meeting about breastfeeding in village	46	4.0%	265	24.3%

		ASBC visited home to give advice on breastfeeding	20	1.7%	138	12.6%
		Participated in at least one group meeting about breastfeeding at the CSPS	169	14.6%	348	31.9%
		Attended a village event organised to discuss breastfeeding	12	1.0%	84	7.7%
OVERALL		Discussed with a health worker 1+ times	754	64.9%	1,097	93.1%
		Received a home visit by an ASBC 1+ times	35	3.0%	244	22.3%
		Participated in at least 1 causerie at a CSPS	227	19.6%	508	46.5%
		Participated in at least 1 causerie in the village	122	10.5%	397	36.4%
		Participated in at least 1 causerie	325	28.0%	676	61.9%
		Attended a village event	12	1.0%	84	7.7%
		Had at least one exposure to one or more of the above	791	68.1%	1,050	96.2%
		Mean exposures* (SD)	2.6 (3.0)		6.6 (4.8)	
		Median exposures* (IQR)	2 (0, 4)		5 (3, 9)	

Table S6: Association between reported exposure to components similar to those in the A&T initiative among mothers living in the intervention arm and the study outcomes

		% Outcome	Risk difference	95% CI		p-value
**Outcome: exclusive breastfeeding among infants <6m (N=552)**						
Discussion with a health worker 1+ times	No	81.8%	Ref.			
	Yes	94.0%	12.2%	-4.8%	29.2%	0.087
Received a home visit 1+ times	No	92.2%	Ref.			
	Yes	96.9%	4.6%	0.3%	8.9%	0.036
Participated in at least 1 causerie at a CSPS	No	91.3%	Ref.			
	Yes	95.7%	4.4%	0.5%	8.3%	0.028
Participated in at least 1 causerie at a village	No	91.6%	Ref.			
	Yes	96.4%	4.7%	-2.0%	11.5%	0.171
Participated in at least 1 causerie	No	89.0%	Ref.			
	Yes	95.4%	6.4%	0.4%	12.4%	0.038
Attended a village event	No	93.2%	Ref.			
	Yes	95.7%	2.5%	-5.1%	10.0%	0.523
Had at least one exposure	No	73.7%	Ref.			
	Yes	94.0%	20.3%	-6.6%	47.2%	0.139
**Outcome: early initiation of breastfeeding among infants <12m (N=1,092)**						
Discussion with a health worker 1+ times	No	16.0%	Ref.			<0.001
	Yes	38.4%	22.3%	12.2%	32.5%	
Received a home visit 1+ times	No	34.3%	Ref.			0.023
	Yes	45.5%	11.2%	1.5%	20.8%	
Participated in at least 1 causerie at a CSPS	No	27.2%	Ref.			<0.001
	Yes	47.8%	20.6%	12.9%	28.3%	
Participated in at least 1 causerie at a village	No	33.7%	Ref.			0.005
	Yes	42.3%	8.6	2.7%	14.6%	
Participated in at least 1 causerie	No	25.4%	Ref.			<0.001
	Yes	42.2%	16.8%	9.3%	24.3%	
Attended a village event	No	36.7%	Ref.			0.159
	Yes	38.1%	1.4%	-13.2%	15.9%	
Had at least one exposure	No	11.9%	Ref.			<0.001
	Yes	37.8%	25.9%	16.9%	34.9%	
**Outcome: gave colostrum among infants <12m (N=1,092)**						
Discussion with a health worker 1+ times	No	93.3%	Ref.			0.379
	Yes	96.2%	2.8%	-3.5%	9.1%	
Received a home visit 1+ times	No	95.6%	Ref.			0.303
	Yes	97.1%	1.5%	-1.3%	4.3%	
Participated in at least 1 causerie at a CSPS	No	93.8%	Ref.			0.017
	Yes	98.4%	4.6%	0.8%	8.4%	
Participated in at least 1 causerie at a village	No	94.7%	Ref.			0.014
	Yes	98.2%	3.6%	0.7%	6.4%	
Participated in at least 1 causerie	No	91.4%	Ref.			0.006

	Yes	98.1%	6.7%	1.9%	11.4%	
Attended a village event	No	95.6%	Ref.			0.051
	Yes	100.0%	4.4%	3.1%	5.6%	
Had at least one exposure	No	88.1%	Ref.			0.157
	Yes	96.3%	8.2%	-3.2%	19.5%	
**Outcome: no pre-lacteal feeds among infants <12m (N=1,092)**						
Discussion with a health worker 1+ times	No	96.0%	Ref.			0.076
	Yes	99.5%	3.5%	-0.4%	7.4%	
Received a home visit 1+ times	No	99.1%	Ref.			0.1278
	Yes	100.0%	0.9%	0.3%	1.6%	
Participated in at least 1 causerie at a CSPS	No	98.6%	Ref.			0.008
	Yes	100%	1.3%	0.4%	2.3%	
Participated in at least 1 causerie at a village	No	99.3%	Ref.			0.939
	Yes	99.3%	-0.0%	-1.0%	0.9%	
Participated in at least 1 causerie	No	98.6%	Ref.			0.221
	Yes	99.6%	1.0%	-0.6%	2.7%	
Attended a village event	No	99.2%	Ref.			0.413
	Yes	100.0%	0.8%	0.2%	1.3%	
Had at least one exposure	No	95.2%	Ref.			0.197
	Yes	99.4%	4.2%	-2.2%	10.6%	
